# Circulating Receptor-Interacting Protein Kinase 3 Are Increased in HBV Patients With Acute-on-Chronic Liver Failure and Are Associated With Clinical Outcome

**DOI:** 10.3389/fphys.2020.00526

**Published:** 2020-06-16

**Authors:** Liwen Chen, Zhujun Cao, Lei Yan, Yezhou Ding, Xinghua Shen, Kehui Liu, Xiaogang Xiang, Qing Xie, Chuanwu Zhu, Shisan Bao, Hui Wang

**Affiliations:** ^1^Department of Infectious Diseases, Ruijin Hospital, Shanghai Jiao Tong University School of Medicine, Shanghai, China; ^2^Intensive Care Unit, The Affiliated Infectious Diseases Hospital of Soochow University, Jiangsu, China; ^3^Department of Infectious Diseases, Ruijin Hospital North, Shanghai Jiao Tong University School of Medicine, Shanghai, China; ^4^Discipline of Pathology, School of Medical Sciences and Bosch Institute, Charles Perkin Centre, University of Sydney, Sydney, NSW, Australia

**Keywords:** HBV, acute-on-chronic liver failure, cell death, RIPK3, necroptosis

## Abstract

**Background and Aims:**

Necroptosis is a newly identified type of cell death with programmed pathways. The current study was performed to investigate necroptosis by measuring its key regulators; receptor interacting protein kinase 3 (RIPK3) and mixed lineage kinase domain-like (MLKL) in patients with Hepatitis B virus (HBV) related acute-on-chronic liver failure (ACLF).

**Methods:**

HBV-related ACLF (HBV-ACLF) patients (*n* = 90), non-ACLF patients without cirrhosis (*N* = 70), patients with cirrhosis (*N* = 40), and healthy controls (HCs; *n* = 70) were enrolled in the study. All patients were subject to serum RIPK3 measurement. Hepatic RIPK3 and MLKL were also determined in the livers of 18 patients and five donors, using immunohistochemistry.

**Results:**

Serum RIPK3 was significantly elevated in HBV-ACLF patients compared to that of non-ACLF patients and the HCs. Serum RIPK3 in ACLF patients at recruitment was significantly higher in non-survivors than those in survivors at the 90-day follow-up. The predictive accuracy of serum RIPK3 at the 90-day outcome was relatively good with an area under the receiver operating curve (AUROC) of 0.72 (*p* < 0.001), similar to that of the model of end-staged liver disease (MELD) score (0.76, *p* < 0.001). The combined use of RIPK3 and MELD score further increased the AUROC to 0.80. The hepatic RIPK3 and MLKL measured by immunohistochemistry, significantly increased in the patients with HBV-ACLF than in the patients without ACLF and the HCs.

**Conclusion:**

Circulating RIPK3 was significantly increased in patients with HBV-ACLF and was associated with a clinical outcome. The improved combined objective scores could offer additional prognostic value in ACLF patients, for physicians with more accurate expectations.

## Introduction

Hepatitis B virus (HBV) infection is still a major challenge, especially in China, leading to unacceptable clinical outcomes e.g., chronic hepatitis B, liver cirrhosis, acute-on-chronic liver failure (ACLF), and hepatocellular carcinoma (HCC; Polaris Observatory Collaborators, 2018). Acute-on-chronic liver failure is an acute deterioration in liver function in the context of chronic liver disease with high mortality within 90 days (Garg et al., 2011; Wang and Zhang, 2013; Jalan et al., 2014). HBV-related ACLF (HBV-ACLF) makes up ∼90% of ACLF in China, which is becoming the major cause of HBV infection-related death (Wang et al., 2014). The outcome of HBV-ACLF is not satisfactory and is characterized by rapid and aggressive progression in different organs and systems. Despite decades of extensive research, the precise pathogenesis of HBV-ACLF remains unclear.

It has been reported that cell death in large numbers is a core event in the progression of liver diseases (Malhi et al., 2010; Cao et al., 2016). Development of CHB is mainly due to persistent hepatic inflammation and hepatocyte death, including apoptosis and necrosis, which ultimately leads to liver failure (Luedde et al., 2014; Cao et al., 2015, 2019a,b). More recently a caspase-independent mode of programmed cell death, termed necroptosis, has been shown to be similar to apoptosis because of the tight regulation by distinct molecules, but it is also characterized by morphological features of necrosis (Declercq et al., 2009; He et al., 2009; Christofferson and Yuan, 2010). Receptor interacting protein kinase 3 (RIPK3; He et al., 2009; Welz et al., 2011) and mixed lineage kinase domain-like (MLKL) pseudokinase are the two key regulators in the development of necroptosis (Sun et al., 2012). Necroptosis is involved in the pathogenesis of inflammation-related diseases, such as inflammatory bowel disease (Pierdomenico et al., 2014), pancreatitis (Wu et al., 2013), and Gaucher disease (Vitner et al., 2014). It is also reported that necroptosis plays a vital role in liver injury in animal models, including ethanol-induced liver injury (Roychowdhury et al., 2013) and acetaminophen-induced liver injury (Ramachandran et al., 2013). However, this controversial finding is also reported by others in pancreatitis (Newton et al., 2016) and acetaminophen-induced liver injury studies (Dara et al., 2015).

Our current study aimed to investigate the involvement of necroptosis in HBV-ACLF.

## Patients and Methods

### Characteristics of Patients

**FIGURE 1:**
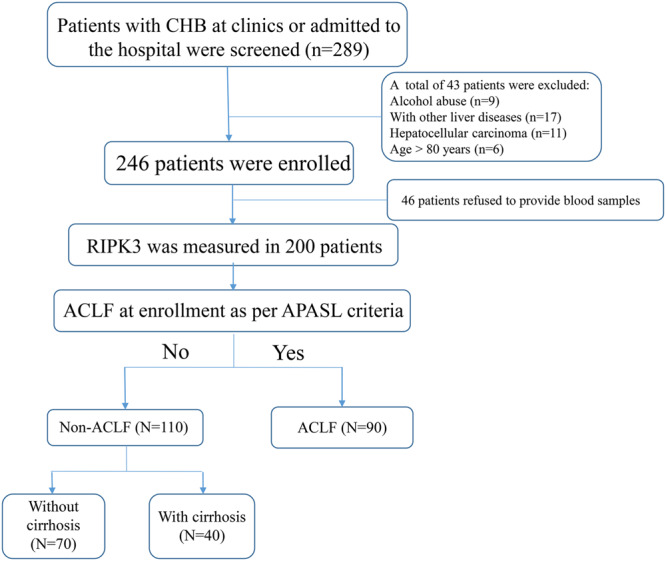
Flow chart of HBV-related patients in the present study.

A total of 289 patients with chronic HBV infection (CHB) were identified from January 2015 to January 2018, retrospectively; whereas 70 healthy controls (HCs) were identified from July 2016 to March 2017 at the Department of Infectious Diseases, Ruijin Hospital, Shanghai, China. The total number subjects were divided into four groups: HBV-ACLF, non-ACLF without cirrhosis, non-ACLF with cirrhosis and HCs. The flow chart of patient enrollment is illustrated in Figure 1.

All HBV-ACLF patients were followed up with for at least 90 days to evaluate the short-term clinical outcomes. The primary endpoint of HBV-ACLF patients was categorized as survivor or non-survivor (underwent liver transplantation or death). CHB was identified as HBV mono-infected with positive hepatitis B surface antigen (HBsAg^+^) for at least 6 months prior to the enrollment in our current study (Terrault et al., 2016). Diagnosis of cirrhosis in CHB was made according to at least one positive result from an ultrasonography, computed tomography, and magnetic resonance imaging.

The diagnostic criteria for HBV-ACLF was based on the consensus recommendations of the *Asian Pacific Association for the Study of the Liver* (APASL; Sarin et al., 2014). Exclusion criteria included: patients with alcoholic liver diseases, non-alcoholic fatty liver diseases, congenital metabolic liver disease, autoimmune liver diseases, evidence of HCC, or age >80 years. In the present study the APASL diagnostic criteria applied for ACLF was due to its suitability for Asians, especially for Chinese ACLF patients. The HBV-ACLF patients were managed according to the APASL consensus recommendations (Sarin et al., 2014).

The present study is in accordance with the Declaration of Helsinki, and has been approved by the Human Ethics Committee, Ruijin Hospital, Shanghai Jiao Tong University School of Medicine. Written informed consent was obtained from the participates.

### Laboratory Assay

Serum biochemical markers included pre-albumin, alanine aminotransferase (ALT), aspartate aminotransferase (AST), total bilirubin, albumin, and creatinine and the international normalized ratio (INR) was routinely measured. Serum HBsAg and hepatitis e antigen (HBeAg) were determined, using commercial enzyme immunoassay kits (AXSYM System; Abbott, Wiesbaden, Germany). The serum HBV DNA level was quantified, using Applied Biosystems PCR system (Prism 7500; Applied Biosystems, Inc., United States), with a lower limit of quantification at 500 IU/mL. All of these measurements were performed routinely by professional technicians at our hospital.

The following formula was used to calculate the Model of end-staged liver disease (MELD) score (Kamath et al., 2001): MELD = 9.57 × LnCreatinine[mg/dL] + 3.78 × LnTotal bilirubin[mg/dL] + 11.2 × LnINR.

### Measurement of Serum RIPK3 Level

Blood samples were collected from patients at enrollment. Serum was separated and stored in −20°C. RIPK3 was measured using a human RIPK3 ELISA kit (CUSABIO, Wuhan, China) (Ma et al., 2018; Sureshbabu et al., 2018; Schenck et al., 2019; Shashaty et al., 2019) following the instructions from the manufacturer.

### Immunohistochemistry and Quantification

Among 23 liver tissues, 10 were from CHB patients undergoing liver biopsies, 8 were from HBV-ACLF patients undergoing liver transplantation and 5 were from healthy liver transplant donors during surgical procedures. Immunohistochemical staining for RIPK3 (Abcam, #ab194699) and MLKL (Abcam, #ab194699) were performed in these 23 liver tissues, according to the experiment protocol as previously described (Lai et al., 2015). Both RIPK3 and MLKL antibodies used for immunohistochemical staining in our study were carefully selected based on applicability, specificity, and also upon validation from other investigators (Mizumura et al., 2014; Wang et al., 2016; Saeed et al., 2019; Xu et al., 2019). A negative control was coupled with the test in which the antibody was substituted by the primary rabbit negative control. The expression of RIPK3 or MLKL was objectively quantified using Image-Pro Plus 7.5 software followed by a macro by presetting the threshold in 10 random fields (400) per stained section. Data were expressed as relative mean density.

### Statistics

Data are presented as the mean ± SD (standard deviation) or medians (25th, 75th percentile) as appropriate. For normally distributed data, an independent-sample t test was used when comparing two groups. For abnormally distributed data, non-parametric statistics were performed, and a Mann–Whitney *U* test was used when comparing two groups. When comparing categorical factors, Chi-square tests were performed. Spearman rank correlation analyses were performed to determine the co-efficient. To evaluate the prognostic value of the combination of RIPK3 and MELD score, we established a novel equation obtained by binary logistic regression as follows: RIPK3-MELD = 0.172 × MELD + 0.001 × RIPK3 − 4.484. A two-tailed *p* < 0.05 was considered statistically significant.

All statistical analyses were performed using SPSS 17.0 statistical software (SPSS Inc, Chicago, IL, United States) and GraphPad Prism 6 (Graph- Pad Software, San Diego, CA, United States).

## Results

### Clinical Characteristics

Patient characteristics of all the study subjects are enumerated in Table 1. The four groups were clinically different, as suggested by all the measured parameters. Compared to other groups, patients with ACLF had remarkably higher levels of liver injury parameters, including ALT, AST, and TB, but lower levels of PAB. Moreover, in serum creatinine, HBV-DNA and coagulation parameters, INR were significantly higher in ACLF than in cirrhotic patients without ACLF (Table 1). All these abnormalities in ACLF contributed to a significantly higher level of MELD score compared to patients without ACLF (27.7 ± 5.8 vs 12.1 ± 4.4, *p* < 0.001).

**TABLE 1:** Patient characteristics at enrollment across different study groups.

		**Non-ACLF without**	**Non-ACLF with**		
**Variable**	**HC (*N* = 70)**	**cirrhosis (*N* = 70)**	**cirrhosis (*N* = 40)**	**ACLF (*N* = 90)**	**P-value**
Male, *n* (%)	28 (40)	54 (77)	25 (63)	77 (86)	<0.001
Age (years)	40.515.6	41.614.5	54.912.1	49.411.7	<0.001
Pre-albumin (mg/L)	266.235.3	255.534.8	84.529.7	56.628.5	<0.001
Alanine aminotransferase (IU/L)	21.57.8	21.98.1	44.841.2	460.5695.1	<0.001
Aspartate aminotransferase (IU/L)	19.96.6	27.016.1	62.855.5	359.8490.6	<0.001
Albumin (g/L)	42.52.4	42.53.7	28.35.1	30.430.5	<0.001
Total bilirubin (μmol/L)	10.04.0	15.27.5	57.575.0	339.7170.6	<0.001
Creatinine (μmol/L)	–	–	67.013.3	84.046.7	0.048
log_10_ HBV DNA (IU/mL)	–	2.60.5	3.11.2	4.11.8	<0.001
International normalized ratio	–	–	1.30.2	2.20.9	<0.001
MELD score	–	–	12.144.4	25.75.8	<0.001

*HC, healthy control, ACLF, acute-on-chronic liver failure, HBV, hepatitis B virus, MELD, model for end-stage liver disease.*

### Necroptosis Associated Proteins Were Increased in HBV-ACLF Patients

**FIGURE 2:**
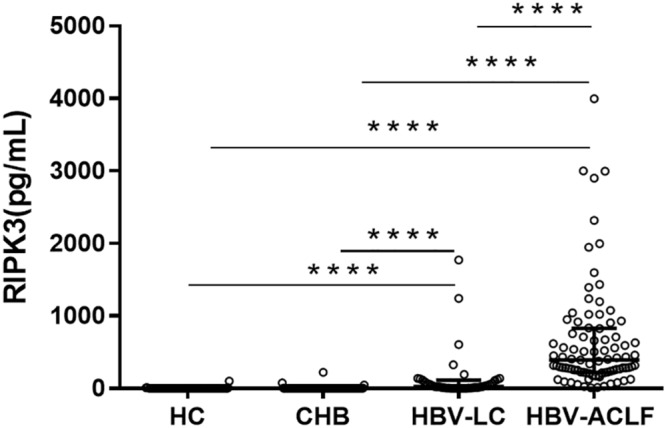
Serum RIPK3 in healthy controls and HBV patients with and without ACLF.

**FIGURE 3:**
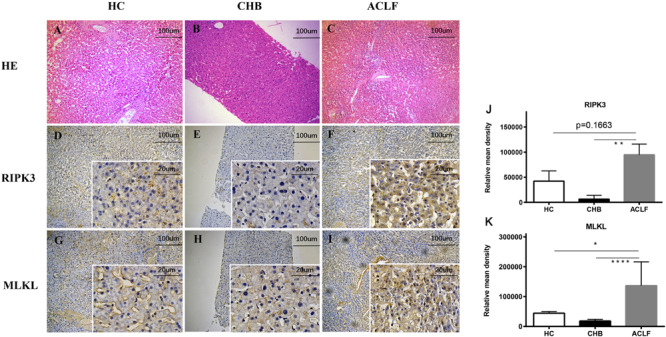
Histopathology of liver tissues with H&E staining from donors (*n* = 5) **(A)**, CHB (*n* = 10) **(B)**, HBV-ACLF (*n* = 8) **(C)** patients (100×). Representative immunohistochemical micrographs of hepatic RIPK3 **(D–F)** and MLKL **(G–I)** in liver tissues of CHB, HBV-ACLF patients and donors. Relative mean density of RIPK3 **(J)** or MLKL **(K)** in immunohistochemical staining was compared across the three groups. *p < 0.05, **p < 0.01, ***p < 0.001.

Serum RIPK3 was measured in our 200 HBV patients and 70 HCs (Figure 2). Serum RIPK3 was undetectable in 94.3 or 92.9% of HCs or non-ACLF patients without cirrhosis, respectively (Figure 2). Cirrhotic patients without ACLF were more likely to have detectable RIPK3, although the level was relatively low. A ∼15-fold higher serum RIPK3 in the HBV-ACLF group compared to those in cirrhotic patients without ACLF was observed (*p* < 0.001; Figure 2). Intrahepatic expression of RIPK3 (*p* < 0.01) and MLKL (*p* < 0.001) with immunohistochemistry staining were both significantly higher in patients with HBV-ACLF than those from CHB patients (Figure 3).

### Serum RIPK3 Was Correlated With Laboratory Markers of Severity of Liver Disease

**FIGURE 4:**
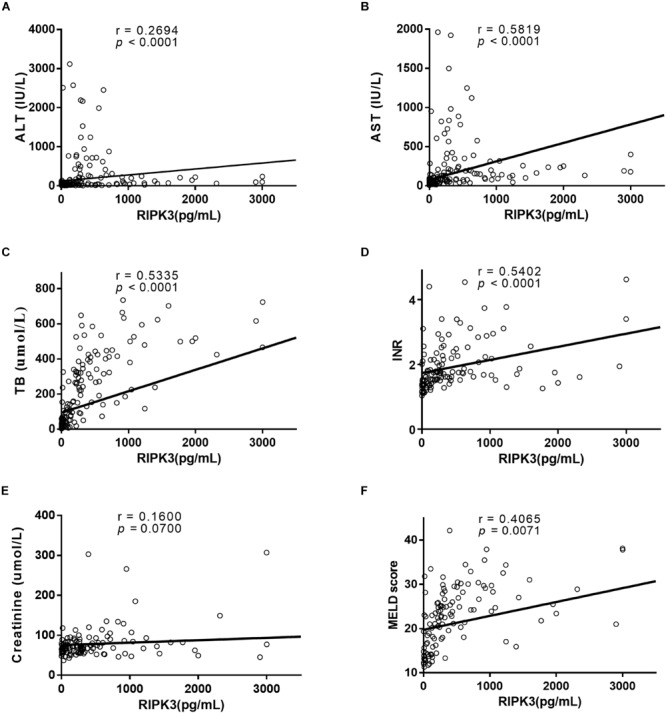
Correlation between serum RIPK3 with ALT **(A)**, AST **(B)**, TB **(C)**, INR **(D)**, Creatinine **(E)**, and MELD score **(F)**.

The correlation between serum RIPK3 and routine laboratory markers was assessed (Figure 4). A positive correlation was observed between serum RIPK3 and ALT or AST (*p* < 0.0001; Figures 4A,B). Serum RIPK3 was also correlated with a severity score of liver disease, MELD score including its parameters TB and INR, but not with serum creatinine (*p* < 0.05; Figures 4C–F).

### Serum RIPK3 Were Higher in HBV-ACLF Survivors Than in HBV-ACLF Non-survivors

**FIGURE 5:**
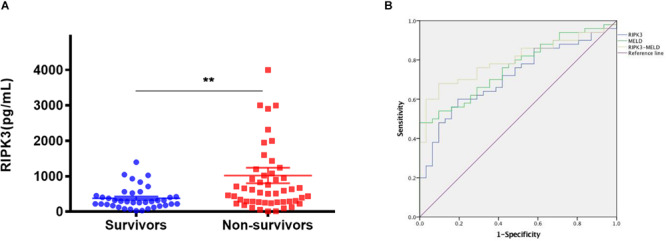
Serum RIPK3 in survivors and non-survivors of HBV-ACLF patients at 90 days **(A)** Sensitivity and specificity for serum RIPK3, MELD score and combination of serum RIPK3 plus MELD score for predicting 90 days’ mortality of HBV-ACLF patients **(B)**. *p < 0.05, **p < 0.01, ***p < 0.001.

Characteristics of ACLF patients at enrollment were compared between survivors and non-survivors (Table 2). Serum total bilirubin, INR and MELD scores were significantly higher in non-survivors than in survivors (all *p* < 0.001). Furthermore, serum in RIPK3 in HBV-ACLF survivors and non-survivors were compared at 90 days. Serum PIPK3 in the non-survivors was 1.9-fold higher than that in the survivors at 90 days (*p* = 0.002, Figure 5A). The prognostic analysis demonstrated that the area under the receiver operating curve (AUROC) of RIPK3 in the prediction of 90-day mortality was 0.715 (*p* < 0.05), which was similar to that of the MELD score (0.763, *p* < 0.05; Figure 5B). The combined use of RIPK3 and MELD scores could further increase the AUROC to 0.80 (Figure 5B).

**TABLE 2:** Baseline patient characteristics in ACLF group according to survival status.

	**Survivor**	**Non-survivor**	
**Variable**	**(*N* = 32)**	**(*N* = 58)**	***p*-value**
Male, *n* (%)	27 (82)	51 (88)	ns
Age (years)	48.110.2	50.112.5	ns
Pre-albumin (mg/L)	60.534.5	54.324.4	ns
Alanine aminotransferase (IU/L)	591.9858.3	384.4575.1	ns
Aspartate aminotransferase (IU/L)	369.4494.9	354.3492.4	ns
Albumin (g/L)	28.75.9	31.438.2	ns
Total bilirubin (μmol/L)	247.3116.4	393.2174.8	<0.0001
Creatinine (μmol/L)	73.416.9	90.156.5	ns
log_10_ HBV DNA (IU/mL)	4.01.9	4.11.8	ns
International normalized ratio	1.80.4	2.41.0	0.0005
MELD score	22.43.6	27.56.1	<0.0001

*ACLF, acute-on-chronic liver failure, HBV, hepatitis B virus, MELD, model for end-stage liver disease.*

## Discussion

ACLF is a life-threatening clinical syndrome with extremely high mortality (Gao et al., 2015); yet much is unknown about the pathogenesis of ACLF. Due to the alteration of intestinal flora, intestinal endotoxin accumulates massively during the development of ACLF, leading to secretion of pro-inflammation cytokines (Kasravi et al., 1996; Li et al., 2010), which subsequently induce various types of cell death.

In our present study, elevated, circulating, and hepatic RIPK3 were correlated with the severity of HBV-related liver diseases, i.e., 90-day mortality. The original source of the elevated serum RIPK3 might derive from damaged hepatocytes, as suggested by the significantly increased hepatic expression of RIPK3 in HBV-ACLF patients. This scenario is consistent with the massive hepatocyte death detected in HBV-ACLF patients (Li et al., 2015). Elevated hepatic RIPK3 might leak from the damaged hepatocytes and subsequently into the circulation. The elevation of RIPK3 might suggest a potential contribution of RIPK3 to necroptosis in HBV-ACLF, due to the involvement of necroptosis in the pathogenesis of inflammation-related diseases (Wu et al., 2013; Pierdomenico et al., 2014) and various liver injury models (Ramachandran et al., 2013; Roychowdhury et al., 2013). However, there is a controversial report on necroptosis in liver diseases, showing that RIPK3 is minimally expressed in the damaged hepatocytes and is not indispensable for the activation of hepatocyte necroptosis (Dara, 2018). Although we are not able to confirm the role of necroptosis in the pathogenesis of ACLF in the current study, we observed that there was a significantly elevated expression of MLKL in the liver of HBV-ACLF patients. MLKL is the downstream protein of RIPK3, acting as an executor of necroptosis. We acknowledge that the increase of RIPK3 or MLKL expression does not reflect the activation of the necroptosis pathway directly, which should be confirmed with the phosphorylation status of both RIPK3 and MLKL. It is currently difficult to detect phosphorylated RIPK3 and MLKL using commercially available antibodies. The precise role of necroptosis in the pathogenesis of ACLF remains to be explored in future studies.

Acute-on-chronic liver failure is a syndrome characterized by acute hepatic decompensation, resulting in liver failure (jaundice and prolongation of the INR). One or more extrahepatic organ failures is/are associated with increased mortality within a period of 28 days and up to 3 months from onset. It has been reported that the mortality of ACLF patients increases sharply within a period of 28 and 90 days (Hernaez et al., 2017). Importantly, our current study demonstrated that serum RIPK3 was able to differentiate between survivors and non-survivors of HBV-ACLF patients at 90 days, i.e., higher serum RIPK3 correlated with a higher number of non-survivors, suggesting that necroptosis contributes to the development of HBV-ACLF which is associated with poor outcomes. This is supported by the positive correlation between serum RIPK3 and MELD scores in our current study, which reflects the severity of HBV-ACLF. In addition, the prognostic accuracy of RIPK3 was similar to the MELD score in predicting 90-day mortality and was significantly increased by the combined use of RIPK3 and MELD. The result was in line with the previous findings in general ICU patients, showing that elevated plasma RIPK3 is associated with organ failure and death (Ma et al., 2018).

Serum RIPK3 in our study was assessed using an established commercial assay that has also been used to investigate necroptosis in sepsis (Ma et al., 2018; Sureshbabu et al., 2018; Schenck et al., 2019; Shashaty et al., 2019). However, it has not been established, beyond a doubt, what is measured in these assays, which means that specific reagents for RIPK3 are not available in the liver and serum. Therefore, it should be interpreted with caution before a well validated assay for serum RIPK3 is fully established. Another limitation of our study is that paired serum and liver tissue samples were not available in our study. Consequently, we were not able to perform a correlation analysis between the circulating level of RIPK3 and hepatic expression of RIPK3. We acknowledge this as a limitation of our study, particularly the phosphorylation of both RIPK3 and MLKL in these patients, but we were not able to clarify this point in the current study. It would be interesting to see whether serum levels of RIPK3 are associated with lactate dehydrogenase, which is a marker of cell necrosis. However, lactate dehydrogenase is not routinely examined in our hospitalized patients. Thus, we are not able to retrospectively perform such tests in the current study but it will be determined in a future study.

## Conclusion

Circulating RIPK3 was significantly increased in patients with HBV-ACLF and was associated with a clinical outcome. The improved combined objective scores can offer additional prognostic value in ACLF patients for physicians with more accurate expectations.

## Data Availability Statement

The datasets generated for this study are available on request to the corresponding author.

## Ethics Statement

The studies involving human participants were reviewed and approved by Human Ethics Committee, Ruijin Hospital, Shanghai Jiao Tong University School of Medicine. The patients/participants provided their written informed consent to participate in this study.

## Author Contributions

HW, SB, and CZ contributed to the study concept and design. LC and LY contributed to the experiment performance. YD, XS, KL, and XX contributed to the patient enrollment and data collection. LC contributed to the manuscript drafting. ZC, QX, CZ, SB, and HW contributed to the critical revision. All authors contributed to the manuscript and approved the submitted version.

## Conflict of Interest

The authors declare that the research was conducted in the absence of any commercial or financial relationships that could be construed as a potential conflict of interest.

## Abbreviations

ACLF: acute-on-chronic liver failure
ALB: albumin
ALT: alanine aminotransferase
APASL: Asian Pacific Association for the Study of the Liver
AST: aspartate aminotransferase
CHB: chronic HBV infection
HBeAg: hepatitis e antigen
HBsAg: hepatitis S antigen
HBV: hepatitis B virus
HCC: hepatocellular carcinoma
HE: hepatic encephalopathy
INR: international normalized ratio
MELD: model of end-staged liver disease
MLKL: mixed lineage kinase domain-like
PAB: pre-albumin
PTA: prothrombin activity
RIPK3: receptor interacting protein kinase 3
TB: total bilirubin.

## References

[B1] Cao Z. Chen L. Li J. Liu Y. Bao R. Liu K. (2019a). Serum keratin-18 fragments as cell death biomarker in association with disease progression and prognosis in hepatitis B virus-related cirrhosis. *J. Viral Hepat.* 26 835–845. 10.1111/jvh.13100 30974482

[B2] Cao Z. Liu Y. Wang S. Lu X. Yin S. Jiang S. (2019b). The impact of HBV flare on the outcome of HBV-related decompensated cirrhosis patients with bacterial infection. *Liver Int.* 39 1943–1953. 10.1111/liv.14176 31206235

[B3] Cao Z. Li F. Xiang X. Liu K. Liu Y. Tang W. (2015). Circulating cell death biomarker: good candidates of prognostic indicator for patients with hepatitis B virus related acute-on-chronic liver failure. *Sci. Rep.* 5:14240. 10.1038/srep14240 PMC4585557 26383863

[B4] Cao Z. J. Li J. Wang Y. Bao R. Liu Y. H. Xiang X. G. (2016). Serum hepatocyte apoptosis biomarker predicts the presence of significant histological lesion in chronic hepatitis B virus infection. *Dig. Liver Dis.* 48 1463–1470. 10.1016/j.dld.2016.07.037 27575659

[B5] Christofferson D. E. Yuan J. (2010). Necroptosis as an alternative form of programmed cell death. *Curr. Opin. Cell Biol.* 22 263–268. 10.1016/j.ceb.2009.12.003 20045303 PMC2854308

[B6] Dara L. (2018). The receptor interacting protein kinases in the liver. *Semin. Liver Dis.* 38 73–86. 29471568 10.1055/s-0038-1629924 PMC5864113

[B7] Dara L. Johnson H. Suda J. Win S. Gaarde W. Han D. (2015). Receptor interacting protein kinase 1 mediates murine acetaminophen toxicity independent of the necrosome and not through necroptosis. *Hepatology* 62 1847–1857. 10.1002/hep.27939 26077809 PMC4681652

[B8] Declercq W. Vanden Berghe T. Vandenabeele P. (2009). RIP kinases at the crossroads of cell death and survival. *Cell* 138 229–232. 10.1016/j.cell.2009.07.006 19632174

[B9] Gao S. Sun F. K. Fan Y. C. Shi C. H. Zhang Z. H. Wang L. Y. (2015). Aberrant GSTP1 promoter methylation predicts short-term prognosis in acute-on-chronic hepatitis B liver failure. *Aliment. Pharmacol. Ther.* 42 319–329. 10.1111/apt.13271 26040771

[B10] Garg H. Sarin S. K. Kumar M. Garg V. Sharma B. C. Kumar A. (2011). Tenofovir improves the outcome in patients with spontaneous reactivation of hepatitis B presenting as acute-on-chronic liver failure. *Hepatology* 53 774–780. 10.1002/hep.24109 21294143

[B11] He S. Wang L. Miao L. Wang T. Du F. Zhao L. (2009). Receptor interacting protein kinase-3 determines cellular necrotic response to TNF-alpha. *Cell* 137 1100–1111. 10.1016/j.cell.2009.05.021 19524512

[B12] Hernaez R. Sola E. Moreau R. Gines P. (2017). Acute-on-chronic liver failure: an update. *Gut* 66 541–553. 10.1136/gutjnl-2016-312670 28053053 PMC5534763

[B13] Jalan R. Yurdaydin C. Bajaj J. S. Acharya S. K. Arroyo V. Lin H. C. (2014). Toward an improved definition of acute-on-chronic liver failure. *Gastroenterology* 147 4–10. 10.1053/j.gastro.2014.05.005 24853409

[B14] Kamath P. S. Wiesner R. H. Malinchoc M. Kremers W. Therneau T. M. Kosberg C. L. (2001). A model to predict survival in patients with end-stage liver disease. *Hepatology* 33 464–470. 10.1053/jhep.2001.22172 11172350

[B15] Kasravi F. B. Wang L. Wang X. D. Molin G. Bengmark S. Jeppsson B. (1996). Bacterial translocation in acute liver injury induced by D-galactosamine. *Hepatology* 23 97–103. 10.1053/jhep.1996.v23.pm0008550055 8550055

[B16] Lai R. Xiang X. Mo R. Bao R. Wang P. Guo S. (2015). Protective effect of Th22 cells and intrahepatic IL-22 in drug induced hepatocellular injury. *J. Hepatol.* 63 148–155. 10.1016/j.jhep.2015.02.004 25681556

[B17] Li H. Xia Q. Zeng B. Li S. T. Liu H. Li Q. (2015). Submassive hepatic necrosis distinguishes HBV-associated acute on chronic liver failure from cirrhotic patients with acute decompensation. *J. Hepatol.* 63 50–59. 10.1016/j.jhep.2015.01.029 25646889

[B18] Li Y. T. Wang L. Chen Y. Chen Y. B. Wang H. Y. Wu Z. W. (2010). Effects of gut microflora on hepatic damage after acute liver injury in rats. *J. Trauma* 68 76–83. 10.1097/ta.0b013e31818ba467 20065761

[B19] Luedde T. Kaplowitz N. Schwabe R. F. (2014). Cell death and cell death responses in liver disease: mechanisms and clinical relevance. *Gastroenterology* 147 765–783. 25046161 10.1053/j.gastro.2014.07.018 PMC4531834

[B20] Ma K. C. Schenck E. J. Siempos I. I. Cloonan S. M. Finkelzstein E. J. Pabon M. A. (2018). Circulating RIPK3 levels are associated with mortality and organ failure during critical illness. *JCI Insight* 3 0–10. 10.1172/jci.insight.99692 PMC6124535 29997296

[B21] Malhi H. Guicciardi M. E. Gores G. J. (2010). Hepatocyte death: a clear and present danger. *Physiol. Rev.* 90 1165–1194. 10.1152/physrev.00061.2009 20664081 PMC2943859

[B22] Mizumura K. Cloonan S. M. Nakahira K. Bhashyam A. R. Cervo M. Kitada T. (2014). Mitophagy-dependent necroptosis contributes to the pathogenesis of COPD. *J. Clin. Invest.* 124 3987–4003. 10.1172/jci74985 25083992 PMC4151233

[B23] Newton K. Dugger D. L. Maltzman A. Greve J. M. Hedehus M. Martin-McNulty B. (2016). RIPK3 deficiency or catalytically inactive RIPK1 provides greater benefit than MLKL deficiency in mouse models of inflammation and tissue injury. *Cell Death Differ.* 23 1565–1576. 10.1038/cdd.2016.46 27177019 PMC5072432

[B24] Pierdomenico M. Negroni A. Stronati L. Vitali R. Prete E. Bertin J. (2014). Necroptosis is active in children with inflammatory bowel disease and contributes to heighten intestinal inflammation. *Am. J. Gastroenterol.* 109 279–287. 10.1038/ajg.2013.403 24322838

[B25] Polaris Observatory Collaborators (2018). Global prevalence, treatment, and prevention of hepatitis B virus infection in 2016: a modelling study. *Lancet Gastroenterol. Hepatol.* 3 383–403. 29599078 10.1016/S2468-1253(18)30056-6

[B26] Ramachandran A. McGill M. R. Xie Y. Ni H. M. Ding W. X. Jaeschke H. (2013). Receptor interacting protein kinase 3 is a critical early mediator of acetaminophen-induced hepatocyte necrosis in mice. *Hepatology* 58 2099–2108. 10.1002/hep.26547 23744808 PMC3791212

[B27] Roychowdhury S. McMullen M. R. Pisano S. G. Liu X. Nagy L. E. (2013). Absence of receptor interacting protein kinase 3 prevents ethanol-induced liver injury. *Hepatology* 57 1773–1783. 10.1002/hep.26200 23319235 PMC3628968

[B28] Saeed W. K. Jun D. W. Jang K. Oh J. H. Chae Y. J. Lee J. S. (2019). Decrease in fat de novo synthesis and chemokine ligand expression in non-alcoholic fatty liver disease caused by inhibition of mixed lineage kinase domain-like pseudokinase. *J. Gastroenterol. Hepatol.* 34 2206–2218. 10.1111/jgh.14740 31132314

[B29] Sarin S. K. Kedarisetty C. K. Abbas Z. Amarapurkar D. Bihari C. Chan A. C. (2014). Acute-on-chronic liver failure: consensus recommendations of the Asian Pacific Association for the Study of the Liver (APASL) 2014. *Hepatol. Int.* 8 453–471. 26202751 10.1007/s12072-014-9580-2

[B30] Schenck E. J. Ma K. C. Price D. R. Nicholson T. Oromendia C. Gentzler E. R. (2019). Circulating cell death biomarker TRAIL is associated with increased organ dysfunction in sepsis. *JCI Insight* 4 0–9. 10.1172/jci.insight.127143 PMC6538332 31045578

[B31] Shashaty M. G. S. Reilly J. P. Faust H. E. Forker C. M. Ittner C. A. G. Zhang P. X. (2019). Plasma receptor interacting protein kinase-3 levels are associated with acute respiratory distress syndrome in sepsis and trauma: a cohort study. *Crit. Care* 23 1–11. 31253195 10.1186/s13054-019-2482-x PMC6599265

[B32] Sun L. Wang H. Wang Z. He S. Chen S. Liao D. (2012). Mixed lineage kinase domain-like protein mediates necrosis signaling downstream of RIP3 kinase. *Cell* 148 213–227. 10.1016/j.cell.2011.11.031 22265413

[B33] Sureshbabu A. Patino E. Ma K. C. Laursen K. Finkelsztein E. J. Akchurin O. (2018). RIPK3 promotes sepsis-induced acute kidney injury via mitochondrial dysfunction. *JCI Insight* 3 1–17. 10.1172/jci.insight.98411 PMC6124406 29875323

[B34] Terrault N. A. Bzowej N. H. Chang K. M. Hwang J. P. Jonas M. M. Murad M. H. (2016). AASLD guidelines for treatment of chronic hepatitis B. *Hepatology* 63 261–283. 10.1002/hep.28156 26566064 PMC5987259

[B35] Vitner E. B. Salomon R. Farfel-Becker T. Meshcheriakova A. Ali M. Klein A. D. (2014). RIPK3 as a potential therapeutic target for Gaucher’s disease. *Nat. Med.* 20 204–208. 10.1038/nm.3449 24441827

[B36] Wang F. S. Fan J. G. Zhang Z. Gao B. Wang H. Y. (2014). The global burden of liver disease: the major impact of China. *Hepatology* 60 2099–2108. 10.1002/hep.27406 25164003 PMC4867229

[B37] Wang F. S. Zhang Z. (2013). Liver: how can acute-on-chronic liver failure be accurately identified? *Nat. Rev. Gastroenterol. Hepatol.* 10 390–391. 10.1038/nrgastro.2013.72 23609466

[B38] Wang L. Wang T. Li H. Liu Q. Zhang Z. Xie W. (2016). Receptor Interacting Protein 3-Mediated Necroptosis Promotes Lipopolysaccharide-induced inflammation and acute respiratory distress syndrome in mice. *PLoS One* 11:e0155723. 10.1371/journal.pone.0155723 27195494 PMC4873150

[B39] Welz P. S. Wullaert A. Vlantis K. Kondylis V. Fernandez-Majada V. Ermolaeva M. (2011). FADD prevents RIP3-mediated epithelial cell necrosis and chronic intestinal inflammation. *Nature* 477 330–334. 10.1038/nature10273 21804564

[B40] Wu J. Huang Z. Ren J. Zhang Z. He P. Li Y. (2013). Mlkl knockout mice demonstrate the indispensable role of Mlkl in necroptosis. *Cell Res.* 23 994–1006. 10.1038/cr.2013.91 23835476 PMC3731568

[B41] Xu H. Du X. Liu G. Huang S. Du W. Zou S. (2019). The pseudokinase MLKL regulates hepatic insulin sensitivity independently of inflammation. *Mol. Metab.* 23 14–23. 10.1016/j.molmet.2019.02.003 30837196 PMC6480316

